# Impaired social decision making in patients with major depressive disorder

**DOI:** 10.1002/brb3.62

**Published:** 2012-07

**Authors:** Hui-jun Zhang, Delin Sun, Tatia M C Lee

**Affiliations:** 1 Laboratory of Neuropsychology, The University of Hong KongHong Kong, China; 2 Laboratory of Cognitive Affective Neuroscience, The University of Hong KongHong Kong, China; 3 The State Key Laboratory of Brain and Cognitive Sciences, The University of Hong KongHong Kong, China; 4 Institute of Clinical NeuropsychologyHong Kong, China

**Keywords:** Affective disorders, altruism, deception, depression, risky decision making, trust

## Abstract

Research on how depression influences social decision making has been scarce. This study investigated how people with depression make decisions in an interpersonal trust-reciprocity game. Fifty female patients diagnosed with major depressive disorders (MDDs) and 49 healthy women participated in this study. The experiment was conducted on a one-to-one basis. Participants were asked to play the role of a trustee responsible for investing money given to them by an anonymous female investor playing on another computer station. In each trial, the investor would send to a participant (the trustee) a request for a certain percentage of the appreciated investment (repayment proportion). Since only the participant knew the exact amount of the appreciated investment, she could decide to pay more (altruistic act), the same, or less (deceptive act) than the requested amount. The participant's money acquired in the trial would be confiscated if her deceptive act was caught. The frequency of deceptive or altruistic decisions and relative monetary gain in each decision choice were examined. People with depression made fewer deceptive and fewer altruistic responses than healthy controls in all conditions. Moreover, the specific behavioral pattern presented by people with depression was modulated by the task factors, including the risk of deception detection and others’ intentions (benevolence vs. malevolence). Findings of this study contribute to furthering our understanding of the specific pattern of social behavioral changes associated with depression.

## Introduction

Mood, whether positive or negative, plays a critical role in interpersonal behavior. Positive affect leads people to interpret external situations optimistically and with trust, and so positive moods may promote altruism and helping behavior. In contrast, negative affect leads people to evaluate social information pessimistically and skeptically (Forgas 2002; Forgas and East 2008; Harlé et al. 2010). Studies have shown that depressed moods magnify self-focus (Isen 2000; McCullough 2000, 2003) and cultivate negative cognitive bias (Elliott et al. 2011). This may explain why studies have consistently linked depression with impaired social functioning (McCullough 2003), that is, the ability to interact with others and adjust behavior in response to changing social contexts (Sanfey 2007; Rilling and Sanfey 2011). Hence, social and interpersonal functioning is an important ingredient of successful interventions for depression (Gotlib et al. 2004; Hammen 2005; Roffman et al. 2005; Cornette et al. 2009). Given that unipolar depression is becoming more prevalent (Song et al. 2008; Gonzalez et al. 2010), it is timely and especially important to understand the influence of depressed moods on social functioning, especially social decision making.

One way to understand social decision making in people with depression is to have them complete tasks that involve cooperation, deception, decisions about risk, and behavior adjustment according to the responses of others. One task that suits these requirements is the trust and reciprocity task first developed by McCabe and colleagues (2001), which we adapted for use in this study. The experimental task of the trust game required each participant (all women) to play the role of a trustee who received an investment from another player (the investor, also a woman [in this study a computer program]). As the investment profited, the trustee was requested by the investor to return a certain portion of the profit to her. Since the investor had no knowledge of the amount of profit, the trustee could decide whether she would return more than (defined as altruistic behavior), equal to (defined as honest behavior), or less than (defined as deceptive behavior) the requested amount.

Navigating the trust and reciprocity task requires decision making to balance risk and reward. But people with depression are less sensitive to the value of rewards and losses (Lerner et al. 2004; Pizzagalli et al. 2008), and this decreased sensitivity may influence their decision making. Indeed, numerous studies have shown that depressed patients fail to maximize the reward value of outcomes in serial decision tasks, seeming to lack the motivation to seek pleasurable stimuli (Lerner et al. 2004; Pizzagalli et al. 2008). Researchers have proposed that this reduced reactivity stems from anhedonia (Henriques and Davidson 2000; Lerner et al. 2004; Pizzagalli et al. 2008). Other studies have proposed a biological explanation for this reduced reactivity, attributing it to dysfunction in the frontocingulate, thereby causing increased cognitive conflict (Knutson et al. 2008; Pizzagalli 2011).

Depressed moods are also related to risk aversion and difficulty making decisions (Must et al. 2006; Nenkov et al. 2008; Smoski et al. 2008; Cella et al. 2010). There are reasons to believe as well that depression also affects altruism and cooperation. Although people with depression report feeling higher levels of guilt and empathic distress (O’Connor et al. 2002), they have weaker intention or ability to help others (O’Connor et al. 2007).

To examine the relationship between depression and social decision making, we tested the behavior of depressed participants in the task game in this study. Because depression is linked with a low intention of helping others as well as low maximizing of benefits to oneself, we hypothesized that people in depressed moods would show less altruistic or deceptive behaviors than people in neutral moods.

## Methods

### Participants

This study was approved by the New Territories West Cluster Clinical and Research Ethics Committee in Hong Kong. Ninety-nine Chinese women aged 21–60 gave written informed consent to participate in the study. Among them, 50 were recruited from both the in-patient and out-patient units of a major psychiatric hospital in Hong Kong. All had been diagnosed with major depressive disorder (MDD) consistent with the diagnostic criteria for MDD and without psychotic features according to the criteria listed in the Diagnostic and Statistical Manual of Mental Disorders, 4th edition (*DSM*-IV, American Psychiatric Association 1994). All had also scored 14 or above on the Chinese version of the Beck depression inventory-II (BDI-II, Chinese Behavioral Sciences Society 2000). The diagnosis was further confirmed by the Chinese version of the Mini International Neuropsychiatric Interview (MINI, Sheehan et al. 1998). Information on comorbidity was obtained from patients’ medical notes and from the Chinese version of the MINI. Patients were excluded if they had histories of physical or psychiatric illnesses—including organic brain disorders, traumatic brain injuries, substance abuse or dependence disorders, psychotic disorders, or mental retardation—that might have affected cognitive functioning. Patients who had received electroconvulsive therapy within six months prior to this study were also excluded from participation. In the MDD group, 28 patients had general anxiety disorder and 34 suffered from dysthymia.

The healthy group consisted of 49 healthy Chinese women free from any history of psychiatric disorders or medical illnesses affecting cognitive functioning and who were recruited from the community. The MDD group and the healthy group were matched for age (MDD group mean ± SD: 45.50 ± 9.28; healthy group: 43.74 ± 8.74) and years of education (MDD group: 8.96 ± 3.39; healthy group: 8.23 ± 2.94, *P*s > 0.1).

### Experimental task

This study's design was adapted from the trust game (McCabe et al. 2001; King-Casas et al. 2005, 2008). Unlike the traditional trust-reciprocity game, this game has all participants play as trustees; in this study, the counterpart of the participant always played the role of investor. Although we used a computer program to play the counterpart, the participants were informed that the investor was a real person, a woman, and that there was a new investor per trial.

The experimental task started with the investor giving the participant (the trustee) *x* amount of money to invest, which appreciated by *N* times. The investor then asked the participant to return a certain percentage of this appreciated amount (*R*) to her, that is, (*R*×*N*×*x*). The participant was supposed to return the exact amount as per the request of the counterpart. The appreciated investment (*N*×*x*) was displayed during the task for the participant's reference. It was made clear to the participant that only she and not the investor would know the exact appreciated amount. At this juncture, the participant was to decide if she would return more (altruistic act), equal to (honest act), or less (deceptive act) than the amount defined by (*R*×*N*×*x*). But if the participant decided to lie to the trustee and this deception was discovered, all money in the trial would be confiscated as punishment. The participant was reminded that she could not pay more than the appreciated investment (*N*×*x*) or less than the amount of investment (*x*).

In each trial, after a pseudorandomized interval meant to mimic a real human decision, the amount of investment (*x*, which was an integer generated from four intervals: 10–20, 30–45, 55–70, and 75–90) was presented on the screen, followed by the appreciated investment (*N*×*x, N* being a rational number selected from four intervals, that is, the investment multiplier: 1–1.2, 1.4–1.6, 2.4–2.6, and 2.8–3). The screen also showed for 2 sec the proportion (*R*) of the investment the trustee should repay the investor and the probability (*P*) that the investor would discover how much the trustee actually paid back. Afterward, the participant was asked to fill in the amount she would like to repay to the investor (M). If the amount of repaid money was larger than that requested, it was considered “altruistic.” But if this amount was less than requested (*R*×*N*×*x*), the participant's response was considered “deceptive.” The participant executed the decision by pressing the spacebar. She then waited for 2 sec to be informed of the money acquired in this trial and whether her deception had been detected by the investor. If the deceptive act was caught, all money acquired in the trial would be confiscated as punishment.

There were three *R* values of requested repayment proportions (20%, 50%, and 80%), which could be defined as “beneficial,”“equal,” and “unfair.” The risk of being detected was defined by two *P* values corresponding to a 25% (low) and a 75% (high) chance of being detected. In total, there were 96 trials corresponding to the conditions combined by the levels of *R, P, N*, and *x* (3 × 2 × 4 × 4 = 96). All trials were presented randomly.

The important dependent measures were frequency of choice and ratio of choice. Frequency of choice meant the number of a type of choice (deceptive or altruistic) relative to all choices made, and indicated the qualitative preference of the participants in social decision making, that is, deception or altruism. The ratio of choice reflected the quantitative preference in choice. If a participant decided to be deceptive, the ratio of choice was the difference between the amount actually repaid and the amount that should be repaid relative to the largest amount that the participant could acquire if she played deception. On the other hand, if the choice was altruism, the ratio of choice was the difference between the amount actually repaid and amount that should be repaid compared with the largest amount that one could repay the investor altruistically.

### Measures

The MINI (Sheehan et al. 1998) is a short structured diagnostic interview, developed jointly by psychiatrists and clinicians in the United States and Europe for *DSM*-IV and ICD-10 (International Classification of Diseases) psychiatric disorders. With an administration time of approximately 15 min, it was designed to meet the need for a short but accurate structured psychiatric interview for multicenter clinical trials, epidemiology studies, and as a first step in outcome tracking in nonresearch clinical settings. Crane and colleagues (2007) argued that MINI is appropriate for use in experimental studies because it requires much less time than the Structured Clinical Interview for the *DSM*-IV (SCID; First et al. 1996). The Chinese version of the MINI was translated by the Taiwanese Society of Psychiatry (Si et al. 2009).

The BDI-II (Beck et al. 1996) is a commonly used assessment of the severity of depression. It is a 21-item self-report inventory measuring the affective, cognitive, and physical symptoms of depression. The Chinese version was translated by the Chinese Behavioral Science Corporation (2000). A few studies have shown that the BDI-II is a valid and reliable assessment tool for Chinese populations (Yeung et al. 2002; Byrne et al. 2004).

### Procedure

Each patient was assessed with the MINI, followed by the BDI-II, to evaluate the severity of her current depressed mood. Healthy controls took only the BDI-II as a preliminary screening. The study was conducted one-to-one in a quiet room at the hospital. Participants then sat in front of a computer, which delivered the experimental task. To make the participants believe that they were playing with real people, a cartoon lasting about 10 sec was presented before the task that informed the participant that the experimental computer was in the process of connecting with the server and the investor. The task lasted about 30 min. Participants were debriefed after the experiment to confirm that they had been actively participating.

### Data analysis

Trials with reaction times exceeding three standard deviations of the mean were excluded. The number of trials excluded was less than 5% of the total trials in each condition for each participant. Repeated-measures analyses of variance (ANOVAs) were then used to analyze the reaction time for all responses, frequencies of deceptive and altruistic responses, and the ratios of deceptive to altruistic responses. The ANOVAs included two within-subject factors: the repayment proportion (*R*, three levels: 20%[low], 50%[equal], and 80%[high]) and the probability that the investor would detect the trustees’ repayment amount (*P*, two levels: 25%[low] and 75%[high]). The differences between the two groups (patients with depression and healthy participants) were then analyzed by between-subject comparison.

## Results

### Frequency of choice for deceptive responses

**Figure 1:**
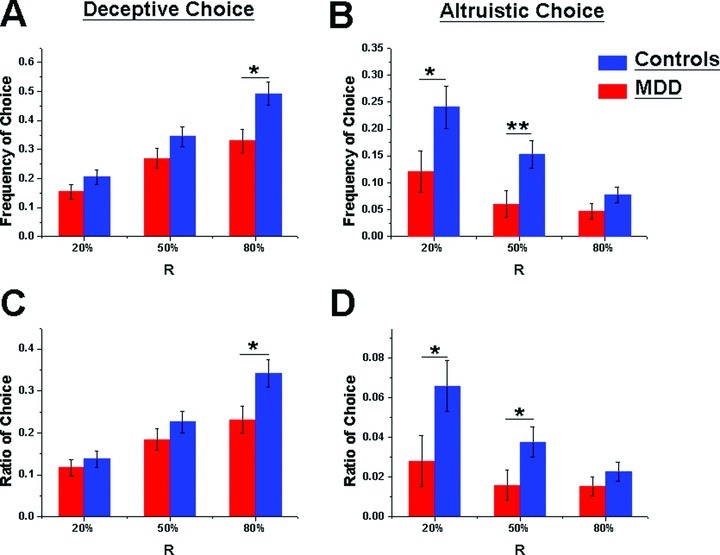
Frequency and ratio of deceptive/altruistic choices as a function of the repayment proportion. Compared with healthy controls, depressed patients made (A) deceptive choices less frequently when the repayment proportion was high (*R*= 80%); (B) altruistic choices less frequently when the repayment proportion was low (*R*= 20%) or medium (*R*= 50%); (C) a smaller ratio of deceptive choices when the repayment proportion was high (*R*= 80%); and (D) a smaller ratio of altruistic choices when the repayment proportion was low (*R*= 20%) or medium (*R*= 50%). R, repayment proportion; Controls, healthy controls; MDD, depressed patients. *P < 0.05; **P < 0.01.

Patients with depression made deceptive responses less frequently (0.25 ± 0.29) than the healthy participants (0.37 ± 0.25), *F*(1, 97) = 4.93, *P*= 0.03, with a significant interaction between repayment proportion and group, *F*(2, 194) = 5.33, *P* < 0.01. Post hoc tests showed that patients with depression also made deceptive decisions significantly less frequently (0.33 ± 0.35) than healthy participants (0.49 ± 0.28) when the repayment proportion was high (*R*= 80%, *F*(1, 97) = 8.02, *P* < 0.01) (Fig. 1A). No significant difference was found between these two groups, however, when the repayment proportion was low or medium (*R*= 20% and 50%, respectively, *P*s > 0.1).

**Figure 2:**
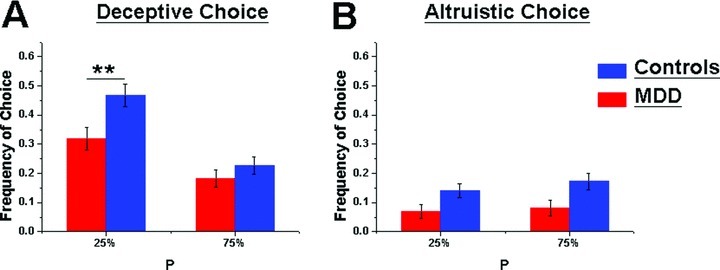
Frequency and ratio of deceptive/altruistic choices as a function of the probability of being detected. Compared with healthy controls, depressed patients made (A) deceptive choices less frequently when the probability was low (*P*= 25%). No significant between-group difference was found for (B) altruistic choices. *P*, Probability; Controls, healthy controls; MDD, depressed patients. **P < 0.01.

An interaction also occurred between risk and group, *F*(1, 97) = 4.90, *P* < 0.03. Post hoc tests showed that patients with depression made deceptive responses less frequently (0.32 ± 0.33) than healthy participants (0.47 ± 0.28) when risk was low (*P*= 25%, *F*(1, 97) = 7.26, *P* < 0.01), but not when risk was high (*P*= 75%, *P* > 0.1) (Fig. 2).

### Frequency of choice for altruistic responses

Patients with depression made altruistic responses (0.08 ± 0.15) less frequently (*F*(1, 97) = 5.46, *P*= 0.02) than healthy participants (0.16 ± 0.25), with a significant interaction between repayment proportion and group, *F*(2, 194) = 3.98, *P*= 0.02. Post hoc tests showed that patients with depression also made altruistic responses less frequently than healthy participants when repayment proportions were low (*R*= 20%, MDD 0.12 ± 0.21 vs. controls 0.24 ± 0.34; *F*(1, 97) = 4.82, *P*= 0.03) or medium (*R*= 50%, MDD 0.06 ± 0.12 vs. controls 0.15 ± 0.24; *F*(1, 97) = 6.79, *P*= 0.01) (Fig. 1B). No significant difference was found between these two groups, however, when the repayment proportion was high (*R*= 80%, *P* > 0.1). The interaction between risk and group was not significant (*F* < 1).

### Ratio of choice for deceptive responses

The interaction between repayment proportion and group was also significant, *F*(2, 194) = 6.19, *P*= 0.002. Post hoc tests showed that patients with depression had a significantly smaller ratio of deceptive responses (0.23 ± 0.28) than healthy participants (0.34 ± 0.24) when the repayment proportion was high (*R*= 80%, *F*(1, 97) = 5.83, *P* < 0.02; Fig. 1C), while no significant difference was found between the two groups when the repayment proportion was low or medium (*R*= 20% or 50%, *P*s > 0.1). There was no significant interaction between risk and group, *F*(1, 97) = 2.85, *P*= 0.094.

### Ratio of choice for altruistic responses

The main effect of group was significant, *F*(1, 97) = 4.24, *P*= 0.04, with the patients’ ratio of altruistic responses (0.02 ± 0.06) being lower than that of the healthy group (0.04 ± 0.08). The interaction between repayment proportion and group was also significant, *F*(2, 194) = 3.37, *P*= 0.04; post hoc results showed that patients with depression repaid a smaller ratio than healthy participants when the repayment proportion was low (*R*= 20%, MDD 0.03 ± 0.07 vs. controls 0.07 ± 0.11; *F*(1, 97) = 4.34, *P*= 0.04) or medium (*R*= 50%, MDD 0.02 ± 0.06 vs. controls 0.04 ± 0.07; *F*(1, 97) = 4.02, *P*= 0.048) (Fig. 1D). There was, however, no significant difference between both groups when the repayment proportion was high (*R*= 80%, *P* > 0.1). The interaction between risk and group was not significant (*F* < 1).

## Discussion

We tested whether depressed people would make more deceptive or altruistic decisions in the modified trust game. The results support our hypotheses that people with depression would in fact make fewer altruistic and fewer deceptive responses.

In this study, executing deceptive or altruistic responses required cognitive affective processing far more complex than that required for simply repaying the suggested amount. For deceptive or altruistic responses, participants needed to consider the risk and payment conjunction and then calculate the difference between the amount of actual repayment and the requested amount before making a decision. Therefore, cognitive load would be much higher if they chose to cheat the investor or to repay an amount different from that of those recruited as reference. People with depression have been widely reported to have compromised cognitive and affective processing (Harvey et al. 2005; Ritchey et al. 2011). Thus, it is logical to reason that these people would simply adhere to the requested payment when preferring to be honest, choose the least repayment when wanting to deceive, or repay as much as possible when deciding to respond altruistically, since other choices would tax their limited cognitive and affective resources. But if this were the case, we should have found a larger ratio of either altruistic or deceptive choices in depressed patients. Instead, compared with healthy participants, people with depression made a smaller ratio of choices on both deceptive and altruistic decisions. The special behavioral patterns of the depressed patients in this study should therefore not have resulted from their limited cognitive or affective resources.

Since the between-group difference was significant in some but not all conditions, this implies that depressed patients were responsive to the varying level of repayment proportion involved in the experiment. Compared with the healthy volunteers, the depressed patients made deceptive responses less frequently and by a smaller ratio only when the repayment proportion was high; they also made altruistic responses less frequently and by a smaller ratio only when the repayment proportion was medium or low. These observations suggest that the behavioral pattern of depressed patients was indeed modulated by the task factor of repayment proportion. The different levels of repayment proportion reflected how benevolent or malevolent the investor was to the participant; in other words, the higher the repayment proportion the investor requested, the less money the participant could retain, and vice versa. In this study, the controls tended to respond altruistically to the investor's benevolent request (low or medium repayment proportion) but deceptively to the investor's malevolent request (high repayment proportion). This is consistent with previous findings that decisions on interpersonal interaction are based on how individuals have treated each other previously (Juliusson et al. 2005; Rilling et al. 2008; Krach et al. 2009). Perceiving a partner's benevolent actions was found to be related with higher activation in the head of the caudate nucleus (King-Casas et al. 2005). Studies have also shown that, compared with normal subjects, depressed subjects had significantly lower mean volumes for the bilateral heads of the caudate nucleus; moreover, such volume reduction was correlated with depression severity (Butters et al. 2009). Depressed patients may thus have difficulty being benevolent because of dysfunctions in the caudate, and therefore fail to respond altruistically. This in turn may prevent them from building advanced relationships with others and lead to their failure in normal social interactions.

Depressed patients also appear to be quite sensitive to negative stimuli (Hamilton and Gotlib 2008; Baert et al. 2010). It is logical to speculate that they harbor strong negative feelings, including pain and anger, with respect to malevolent treatment. Indeed, previous studies have shown that people rejected (malevolent response) an unfair offer (malevolent requirement) with anger (Pillutla and Murnighan 1996), suggesting that the negative emotion (i.e., anger) plays an important role in reacting to malevolence. Therefore, the fact that the depressed patients in this study made fewer malevolent (i.e., deceptive) responses might be attributed to their difficulty in converting the emotion of anger into an actual action of revenge. This opinion is consistent with the findings of a recent study by Harle et al. (2010) that depressed individuals reported a more negative emotional reaction (anger, disgust, and surprise) to unfair offers, but still accepted significantly more of these offers than did the controls. Malevolence has been previously reported to be related to higher activation in the anterior insula. Furthermore, this increased activation predicted participants’ decisions to make a malevolent response (e.g., rejecting offers) (Sanfey et al. 2003). The anterior insula may thus be important in converting the feeling of anger into a malevolent response to others’ malevolent actions. A recent study showed that, compared with healthy controls, major depressed patients showed significantly reduced neural activity, particularly in the bilateral anterior insula (Wiebking et al. 2010). In line with this thought, depressed patients facing a malevolent requirement may find transforming the feeling of anger into a response of revenge (deceptive repayment) rather challenging. Revenge against a malevolent requirement has been proposed to serve as a fundamental adaptive mechanism by which people assert and maintain a social reputation (Nowak et al. 2000). Therefore, depressed patients in normal social life may fail to adjust to others’ malevolence by revenge and fall deeply into the mire of negative feelings, which may in turn further enhance the severity of their symptoms.

Destoop et al. (2012) investigated decision making in people with depression using a modified ultimatum game paradigm. Participants were asked to play as responder and then proposer against the same partner. The results showed that depressed patients in the role of responder accepted both fair and unfair offers. Following our speculation above, depressed patients in Destoop et al.'s study might have found it difficult to fight back the unfair offers. Future studies may contribute to clarify the mechanisms of this particular behavioral presentation of people with depression.

Additionally, only when the risk of being detected was low did the patients in the present study make fewer deceptive responses than the controls. That is, the controls tended to lie more frequently when the risk of being detected was low because they would be exposed and punished less frequently in this condition. Compared with the healthy participants, depressed patients might have tried to avoid risky decision making (deception) even when the risk was low. This idea is supported by a study by Smoski et al. (2008), who observed that depressed patients performed better than controls in the Iowa gambling task, a finding that could be understood only from the perspective that depressed patients were risk avoidant.

In sum, the behavior of people with depression of being relatively less altruistic as well as less deceptive than their healthy counterparts reflects their tendency to be very self-focused. Depressed patients may have difficulty in integrating information of both risk and others’ intentions into social decision making. Their impaired interpersonal interaction could have a biological basis, which would be worth further exploring in future studies. Indeed, both animal (Grippo et al. 2007, 2011) and human (Hinojosa et al. 2011) studies have shown that social isolation is a predictor of depression. Our results provide further evidence that depressed patients behave in a particular way that isolates them from other people in social interactions. This specific behavioral pattern might contribute to their further social isolation and may thus intensify their depression.

This study investigated the decision making of depressed patients in interpersonal interactions in an economic exchange game. This lab task did not reflect a real social interaction. Rather, it was only a very simplified model of social behavior that failed to capture other important domains of social interaction, for example, communication through verbal language (Duff et al. 2009), nonverbal language (Brune et al. 2009), facial expressions (Mojzisch et al. 2006), and eye contact (Voncken et al. 2006). Future studies may advance our understanding of the social behaviors of depressed patients by involving more factors of social interaction. Pairing behavioral with neuroimaging studies in the future could also help unravel the neural mechanisms underlying the behaviors. Moreover, Fujiwara (2009) have recently shown that people who make altruistic financial contributions to individuals other than family members may be at risk of developing major depression. Therefore, it is difficult to conclude that the depressed patients’ special behavioral pattern in social decision making is the consequence of their mental disorder. Future longitudinal studies may contribute to addressing the causal relationship between major depression and abnormal choices in social decision making.

## Conclusion

People with depression made fewer deceptive and altruistic decisions relative to their healthy counterparts. The specific behavioral pattern presented by people with depression was modulated by the task factors, including the risk of deception detection and others’ intentions (benevolence vs. malevolence). These results contribute to furthering our understanding of the specific pattern of social behavioral changes associated with depression. The findings of this study should prompt further experimentation to identify effective interventions for remediating the social behavioral deficits associated with depression in order to promote a quality social life and rewarding social integration for people with depression.
